# Liver-Directed Therapy for Neuroendocrine Metastases: From Interventional Radiology to Nuclear Medicine Procedures

**DOI:** 10.3390/cancers13246368

**Published:** 2021-12-19

**Authors:** Roberto Luigi Cazzato, Fabrice Hubelé, Pierre De Marini, Eric Ouvrard, Julien Salvadori, Pietro Addeo, Julien Garnon, Jean-Emmanuel Kurtz, Michel Greget, Luc Mertz, Bernard Goichot, Afshin Gangi, Alessio Imperiale

**Affiliations:** 1 Interventional Radiology, University Hospitals of Strasbourg, Strasbourg University, 67000 Strasbourg, France; robertoLuigi.cazzato@chru-strasbourg.fr (R.L.C.); pierredemarini@chru-strasbourg.fr (P.D.M.); julien.garnon@chru-strasbourg.fr (J.G.); michel.greget@chru-strasbourg.fr (M.G.); afshin.gangi@chru-strasbourg.fr (A.G.); 2 Oncology, Institut de Cancérologie de Strasbourg Europe (ICANS), Strasbourg University, 67200 Strasbourg, France; je.kurtz@icans.eu; 3 Nuclear Medicine and Molecular Imaging, Institut de Cancérologie de Strasbourg Europe (ICANS), University Hospitals of Strasbourg, Strasbourg University, 67200 Strasbourg, France; f.hubele@icans.eu (F.H.); e.ouvrard@icans.eu (E.O.); 4 Radiophysics, Institut de Cancérologie de Strasbourg Europe (ICANS), 67200 Strasbourg, France; j.salvadori@icans.eu; 5 Hepato-Pancreato-Biliary Surgery and Liver Transplantation, University Hospitals of Strasbourg, 67200 Strasbourg, France; pietrofrancesco.addeo@chru-strasbourg.fr; 6 Radiophysics, University Hospitals of Strasbourg, 67000 Strasbourg, France; luc.mertz@chru-strasbourg.fr; 7 Internal Medicine, Diabetes and Metabolic Disorders, University Hospitals of Strasbourg, Strasbourg University, 67200 Strasbourg, France; bernard.goichot@chru-strasbourg.fr; 8 School of Biomedical Engineering and Imaging Science, King’s College London, Strand, London WC2R 2LS, UK; 9 Molecular Imaging—DRHIM, IPHC, UMR 7178, CNRS/Unistra, 67037 Strasbourg, France

**Keywords:** neuroendocrine, liver metastasis, liver-directed therapy, interventional radiology, nuclear medicine, radioembolization

## Abstract

**Simple Summary:**

Approximately 80% of metastatic patients with gastroenteropancreatic neuroendocrine neoplasms (GEP-NENs) have secondary hepatic lesions, and in approximately 50% of cases, the liver is the only metastatic site. In patients with hepatic metastases from NENs for whom surgical treatment is contraindicated (high liver involvement, inaccessible localizations), percutaneous or intra-arterial treatments are safe and effective options to achieve disease control. In selected patients, liver-directed therapy could allow the improvement of clinical symptoms and biological abnormalities related to tumor secretion. However, toxicity and quality of life are also important elements in therapeutic decisions and must be considered for each single patient. Prospective studies are necessary to define the best treatment combination, including systemic and local options, to improve patient management.

**Abstract:**

Neuroendocrine neoplasms (NENs) are rare and heterogeneous epithelial tumors most commonly arising from the gastroenteropancreatic (GEP) system. GEP-NENs account for approximately 60% of all NENs, and the small intestine and pancreas represent two most common sites of primary tumor development. Approximately 80% of metastatic patients have secondary liver lesions, and in approximately 50% of patients, the liver is the only metastatic site. The therapeutic strategy depends on the degree of hepatic metastatic invasion, ranging from liver surgery or percutaneous ablation to palliative treatments to reduce both tumor volume and secretion. In patients with grade 1 and 2 NENs, locoregional nonsurgical treatments of liver metastases mainly include percutaneous ablation and endovascular treatments, targeting few or multiple hepatic metastases, respectively. In the present work, we provide a narrative review of the current knowledge on liver-directed therapy for metastasis treatment, including both interventional radiology procedures and nuclear medicine options in NEN patients, taking into account the patient clinical context and both the strengths and limitations of each modality.

## 1. Introduction

Neuroendocrine neoplasms (NENs) are rare and heterogeneous epithelial tumors with neuroendocrine differentiation that most commonly arise from the gastroenteropancreatic (GEP) system. GEP-NENs account for approximately 60% of all NENs, and the small intestine and pancreas represent two most common sites of primary tumor development. The annual age-adjusted incidence and prevalence of NENs are progressively increasing, mainly due to the improved sensitivity of modern diagnostic techniques, as suggested by older autopsy studies showing that most cases were not diagnosed during the patient’s lifetime [1,2,3,4].

NENs can be secretory and cause symptoms related to tumor secretion. However, patients are often asymptomatic or present with general symptoms, nonspecific biological abnormalities, or incidental findings on imaging studies. Usually, clinical symptoms are associated with advanced disease and the presence of systemic metastatic spread. Intestinal obstruction or incidental detection of liver metastases commonly lead to the diagnosis of NENs. To optimize the therapeutic strategy, the patient should be presented to a multidisciplinary board that includes liver surgeons, oncologists, radiation oncologists, endocrinologists, radiologists, interventional radiologists, and nuclear physicians with substantial experience in NENs. The only curative treatment for patients with NENs is surgery, including resection of the primary tumor [5,6,7]. However, even in advanced disease, long patient survival is not uncommon, mainly because of the slow evolution of many of these tumors [8]. While cure is the goal for localized tumors, it is rarely achievable in patients with metastatic disease, and preservation of quality of life remains essential. Control of the secretory syndrome, treatment of complications, and consideration of toxicity of therapies are critical. Thus, locoregional treatments will be proposed not only for antitumor purposes in patients with proven disease progression or high tumor volume but also to frequently control secretory syndrome in cases of ineffectiveness of systemic treatment, such as cold somatostatin analogs.

In the present work, we provide a narrative overview of the current knowledge of liver-directed therapy for the treatment of metastases in patients with NENs, including both interventional radiology procedures and nuclear medicine options, taking into account the patient’s clinical context and both the strengths and limitations of each modality.

## 2. NEN Liver Metastases

**Figure 1:**
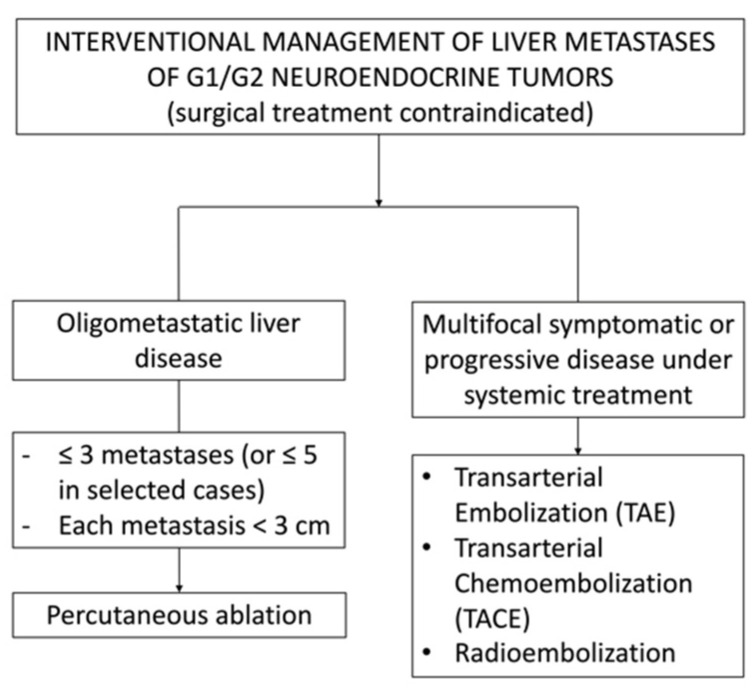
Simplified therapeutic algorithm for the interventional management of liver metastases in patients with G1/G2 NENs without surgical indication.

Approximately 80% of metastatic patients have secondary liver lesions. Furthermore, in approximately 50% of patients, the liver is the only metastatic site [9]. The presence of liver metastases, the degree of secondary liver invasion, and hepatocellular failure due to tumor infiltration of the liver parenchyma are critical factors in assessing prognosis and optimizing therapeutic management, particularly the choice of regional treatment such as surgery or direct liver treatments. Thus, it seems clear that therapeutic strategies vary depending on the degree of hepatic metastatic invasion. In oligometastatic patients, curative approaches such as surgery or percutaneous ablation may be indicated. Conversely, for patients with multiple, bilateral, and unresectable metastases, a strategy to increase life expectancy and quality of life should be discussed [10], by taking into account the control of symptoms, as well as the risks and side effects of each therapeutic option (Figure 1). In this context, locoregional approaches should be balanced with well-tolerated systemic treatments such as somatostatin analogues, generally proposed as first-line treatment, and other more toxic options such as chemotherapy, tyrosine kinase inhibitor (e.g., sunitinib), or mTor pathway inhibitor (e.g., everolimus). Regional treatments may be also used to delay or to provide “holidays” from these systemic treatments.

Liver-only metastatic disease from NENs could represent a potential indication for liver transplantation (LT). However, the place of LT for liver metastases remains debated mainly because of (1) the absence of randomized studies, (2) the high rate of recurrence after LT (60%) for this indication, (3) the long and indolent biologic behavior of metastatic well-differentiated NENs, (4) the reported efficacy of non-surgical therapy for metastatic NENs including new biological systemic treatment, and (5) the context of organ shortage [11]. From the analysis of the literature, current recommendations for LT in patients with metastatic NENs include patient younger than 55 years, primary tumor drained by the portal system (pancreas and intermediate gut) already removed with a curative resection before LT, less than 50% involvement of liver parenchyma, stable disease for at least 6 months before LT, Ki67 < 10%, low-grade and well-differentiated tumor (G1, G2), absence of extrahepatic disease, and no extrahepatic combined resection [12,13,14]. However, those criteria remain to be validated in larger studies.

Nonsurgical locoregional treatments of liver metastases from NENs mainly include percutaneous ablation and endovascular treatments, which allow local disease control by targeting liver metastases individually or according to a segmental or lobar distribution, respectively. Currently, in patients with grade 1 and 2 NENs, only a few treatments have been evaluated in randomized controlled trials. Most of the available studies are retrospective and involve a limited number of patients [15]. In any case, it should be emphasized that among the different techniques proposed for the detection and treatment of liver metastases, the optimal method most likely depends on the patient’s clinical history, the available equipment, and the experience of the medical teams.

## 3. Current Imaging for Management of NEN Liver Metastases

To identify NEN patients with inoperable disease, it is essential to define the real extent of metastatic spread, particularly in the liver. With this aim, medical imaging plays a crucial role in assessing the locoregional extension and metastatic spread. In the evaluation of liver metastases from GEP-NENs, a variable combination of ultrasound (US), computed tomography (CT), magnetic resonance imaging (MRI), and multitracer positron emission tomography (PET) is typically adopted, taking into account the clinical context and both the strengths and limitations of each diagnostic modality.

The available imaging modalities frequently miss hepatic metastases sized less than 5 mm [16]. MRI is a very sensitive technique for the detection of liver metastases and is considered more accurate than both US and CT [16]. On multiphasic contrast-enhanced MR (or CT) images, hepatic lesions are usually hypervascular in the arterial phase and demonstrate washout in the portal and late phases [17,18]. Multiphasic MRI with fat-suppressed contrast-enhanced T1-weighted imaging provides the best accuracy [18,19]. Typically, NEN lesions show T2 hyperintensity and T1 hypointensity. The use of diffusion-weighted imaging (DWI) and apparent diffusion coefficients (ADCs) definitively improves liver lesion detection, thus making MRI more advantageous than CT in terms of diagnostic accuracy [20]. Moreover, given the expected long patient survival and their relatively young age, MRI seems even more adapted than CT due to the lack of ionizing radiation. Liver-specific contrast media seems to be a promising alternative to gadolinium-based agents and are already used in clinical practice [21].

Nuclear medicine imaging for NENs is mainly based on specific molecular characteristics of the neuroendocrine phenotype, such as the ability to take up and decarboxylate amine precursors (^18^F-fluorodihydroxyphenylalanine (^18^F-DOPA)) and the overexpression of somatostatin membrane receptors (radiolabeled-DOTA-peptides). ^18^F-DOPA PET has excellent diagnostic performance in patients with low-grade midgut NETs [22]. In this clinical setting, ^18^F-DOPA PET is indicated for tumor localization and staging, particularly in symptomatic patients (carcinoid syndrome) [23]. Concerning the somatostatin receptor-based imaging, one gamma camera radiopharmaceutical is commercially available (^111^In-pentetreotide; Octreoscan™; Mallinckrodt, NL, USA) and technetium-99m-labeled tracers have been described, using both octreotide and octreotate. In the last decade, a novel class of radiopharmaceuticals based on octreotide derivatives labeled with the positron emitting radionuclide gallium-68 has emerged as the current gold standard for well-differentiated tumors. Compared with ^111^In-pentetreotide, their better type-2 receptor affinity profile, combined with the physical advantages of current clinical PET cameras over gamma cameras, allows detection of smaller lesions and lesions with even low or moderate receptor expression, resulting in a higher diagnostic sensitivity [24]. Glycolytic metabolism could also be investigated using ^18^F-fluorodeoxyglucose (^18^F-FDG), a diagnostic molecular probe widely used in oncologic clinical practice. ^18^F-FDG PET is considered the preferred technique for the identification and staging of high-grade tumors [24]. Usually, a value of Ki67 above or equal to 10% is empirically considered as the cutoff to propose ^18^F-FDG PET for well-differentiated tumors [25]. ^18^F-FDG PET seems to have a potential value for prognostic stratification in patients with NETs [26]. Moreover, some evidence indicates that ^18^F-FDG PET plays a role in predicting response to ^177^Lu-PRRT monotherapy, allowing the identification of patients with grade 1 and 2 metastatic NETs who might benefit from more intensive therapy protocols [27]. Usually, a personalized nuclear medicine investigation will be tailored for each patient, considering both clinical symptomatology and tumor characteristics (mainly tumor differentiation and grade) [24].

Anatomic and functional imaging are usually combined in “hybrid” modalities as PET/CT devices. Currently, PET/MRI hybrid systems are also available and may contribute to increasing diagnostic accuracy, although their current accessibility in clinical practice remains limited.

## 4. Imaging Guidance for Percutaneous Interventional Procedures

Percutaneous ablation may be provided with multiple modalities of imaging guidance, including US, CT, MRI, and PET/CT. The most common modality is US alone or in combination with CT. When CT guidance alone is used, contrast-enhanced CT acquisitions obtained at the beginning of the procedure are generally used to plan needle targeting due to the low conspicuity of the metastases on unenhanced CT images. To facilitate such a task, liver immobilization obtained with jet ventilation may also be applied [28]. Another elegant alternative is represented by MRI guidance, which may often grant spontaneous tumor visualization due to the high intrinsic soft-tissue resolution and multiplanar 3D MRI-fluoroscopy facilitating needle navigation [29].

**Figure 2:**
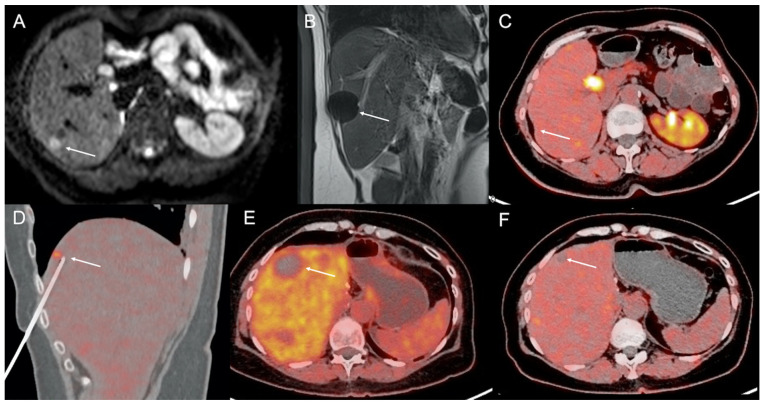
A 55-year-old woman with grade 1 ileal NEN liver metastases and a previous history of MRI-guided liver metastasis ablation. MRI diffusion-weighted axial image showed a target liver metastasis (arrow) of segment VI (**A**), effectively treated with percutaneous MRI-guided cryoablation (**B**, arrow: iceball), which resulted in a complete retraction of the ablation site without any pathologic ^18^F-DOPA uptake during follow-up PET/CT (**C**, arrow). Four years after the first treatment, the patient underwent ^18^F-DOPA PET/CT-guided radiofrequency ablation of a 10 mm liver metastasis (**D**, arrow) of the IV liver segment. Immediate postablation ^18^F-DOPA PET-CT showed complete tumor destruction without residual ^18^F-DOPA uptake (**E**, arrow). ^18^F-DOPA PET/CT performed 24 months later showed limited parenchymal scarring without pathologic ^18^F-DOPA uptake (**F**, arrow).

Currently, the wide availability of hybrid diagnostic systems offers exciting prospects for targeting tumors that are not well visualized by radiological imaging techniques. When none of the aforementioned imaging modalities allows safe and effective tumor visualization, the interventional radiology procedure may be proposed under PET/CT guidance, which allows long-lasting (for several minutes) “metabolic” visualization of the target tumor [30,31] and image-guided invasive diagnostic and therapeutic procedures such as percutaneous biopsy and radiofrequency ablation of liver metastases [32,33]. PET/CT-guided biopsy may also be useful to confirm metabolic findings (potentially influencing therapeutic strategy) when conventional imaging fails to show morphologic abnormalities (Figure 2). Nevertheless, it should be noted that PET/CT is highly irradiating both for the patient and the operator. Therefore, such an option should still be reserved for a few selected cases [34].

## 5. Percutaneous Ablations

Percutaneous ablation relies on the direct application of thermal or nonthermal energy to the target tumor to achieve its destruction [35]. The goal of such treatment is to ideally provide complete destruction of the target metastasis along with some surrounding normal tissue to prevent any residual or recurring tumor due to microscopic tumor deposits [35]. Several ablation modalities are now available to perform the percutaneous treatment of tumors; nevertheless, the most common ablation modalities used for liver tumors, including metastases from NENs, are laser ablation (LA), radiofrequency ablation (RFA), microwave ablation (MWA), and irreversible electroporation (IE). The first three modalities are based on thermal energy, and the last modality relies on the delivery of nonthermal energy. In practice, LA, RFA, and MWA aim at creating extreme temperature increases (>55 °C) in the target tumor, resulting in protein denaturation with subsequent coaugulative necrosis [35]. On the other hand, IE applies a high-voltage electric current to the tumor to generate multiple holes (“pores”) on the external cellular membrane. Therefore, physiological ionic homeostasis across the cellular membrane is lost, which will finally result in cellular death [35]. Each of the aforementioned techniques presents some advantages and disadvantages (Table 1), and operators should choose the most adapted technique based on the specific features of the case, local experience, and equipment availability. From a clinical point of view, percutaneous ablation in patients with hepatic metastases from NENs is applied with the intent of achieving (a) effective local tumor control in patients with oligometastatic or oligoprogressive liver disease and (b) effective control in symptomatic patients presenting with carcinoid syndrome (Table 1). It should be noted that in the last years, percutaneous ablation has been increasingly used for the treatment of liver metastases, including those from NENs; and among all the aforementioned ablation techniques, RFA and MWA seem to currently play the most relevant role [36].

### 5.1. Laser Ablation (LA)

LA provides small-sized (generally less than 2 cm) ablation zones when one single fiber is used. Moreover, LA is relatively cheap and widely available and requires small (18 G) percutaneous accesses through which the laser fiber is coaxially deployed [35]. In a retrospective series including 189 liver metastases (mean diameter 17.9 mm) from NENs in 21 patients, Sartori et al. [26] reported a technical efficacy of 100%, a primary efficacy rate of 94.7%, and a secondary efficacy rate of 100% at a median follow-up of 39 months (range 12–99). Complete symptom relief was obtained in all symptomatic patients (n = 13). The 1-, 3-, and 5-year survival rates were 95%, 66%, and 40%, respectively. Interestingly, overall survival was higher for patients with Ki-67 expression ≤ 7% than for those with Ki-67 > 7%. Moreover, the procedure proved to be highly safe, with only one (0.53%) grade 4 complication being reported.

### 5.2. Radiofrequency Ablation (RFA)

Compared with LA, RFA provides larger ablation zones (approximately 3 cm in size) when one single electrode is activated; furthermore, even larger ablation zones (up to 4–4.5 cm) can be achieved with clusters of electrodes (generally up to three) activated simultaneously [35]. In addition, RFA represents one of the oldest ablation techniques available on the market, which accounts for the most robust literature on the subject. Nevertheless, RFA effectiveness in achieving a large ablation zone may be limited by the heat-sink effect [37].

In a recently published systematic review including eight studies and 301 patients (54% symptomatic) with liver metastases from NENs [38], 92% of symptomatic patients reported symptom improvement following RFA (performed percutaneously in 156 patients), with a median duration of symptom improvement ranging between 14 and 27 months. Although symptom recurrence was somehow common (63–87%), it should be noted that one of the main advantages of percutaneous treatments is the possibility of repeating the treatment several different times, even on the same target.

### 5.3. Microwave Ablation (MWA)

**Figure 3:**
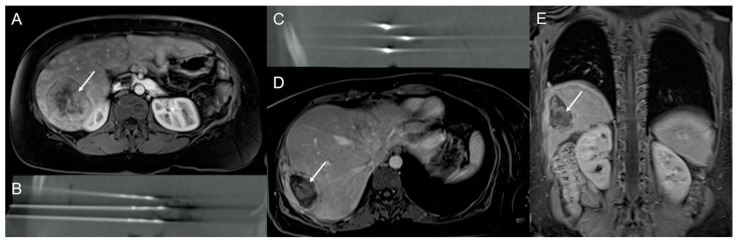
A 39-year-old woman with multiple liver metastases from a grade 2 neuroendocrine tumor that previously underwent systemic treatments. (**A**) Axial contrast-enhanced T1-weighted MRI image showing a single metastasis of 6.5 cm (arrow) of the right liver lobe, which was not responsive to previous therapies. According to a multidisciplinary board recommendation, tumors were treated by microwave ablation performed with a multiantenna approach (**B**,**C**), complicated by postablation bleeding and local infection treated by percutaneous embolization and drainage, respectively. Axial (**D**) and coronal (**E**) contrast-enhanced T1-weighted MRI images obtained at the 9-month follow-up showing a shrunken residual necrotic area (arrows).

MWA is intended to provide very large ablation zones (up to 4 cm with one single antenna and up to 5–6 cm with a cluster of three antennas activated simultaneously) in a very short time interval (generally 5–10 min) [39,40] (Figure 3). Moreover, compared with RFA, MWA is expected to suffer less from the heat-sink effect [41].

In a recently published retrospective series [42] including 50 patients with 166 NEN liver metastases treated with MWA, 41 (82%) were treated with a minimally invasive approach, and 22 (44%) were treated with MWA and concomitant surgical resection. A total of 70% of patients were treated with curative intent, with a 77% (27/35) success rate. On the other hand, there were 40% (20/50) symptomatic patients, and MWA allowed a clinical improvement in 19/20 (95%). Recurrence-free and overall survival rates at 1 and 5 years were 86% and 28%, and 94% and 70%, respectively (median follow-up 32 months, range 0–116 months).

### 5.4. Irreversible Electroporation (IE)

IE results in a relatively small ablation zone (up to 2–3 cm) when a cluster of three to four electrodes is activated. Nevertheless, the main advantage of such ablation modality is that it spares the collagenous structures supporting the biliary ducts and main liver vessels; therefore, it is particularly indicated for the treatment of very central liver tumors [43,44,45]. Nevertheless, IE should be carefully applied in patients with heavy cardiac history since cases of IRE-induced arrythmia have been reported [46].

In a prospective single-center study [47] including 65 malignant liver tumors (including three NENs metastases; median tumor diameter 2.4 cm) in 34 patients undergoing IRE, 3-, 6-, and 12-month local recurrence-free survival rates were 87.4%, 79.8%, and 74.8%, respectively. The median time to progressive disease was 15.6 months, and the overall complication rate was 27.5%, with six major (one intraperitoneal bleeding, one portal vein thrombosis, and four liver abscesses) and eight minor (six and two cases of liver hematoma and pneumothorax, respectively) events.

## 6. Endovascular Treatments

### 6.1. Transarterial Embolization (TAE) and Transarterial Chemoembolization (TACE)

**Figure 4:**
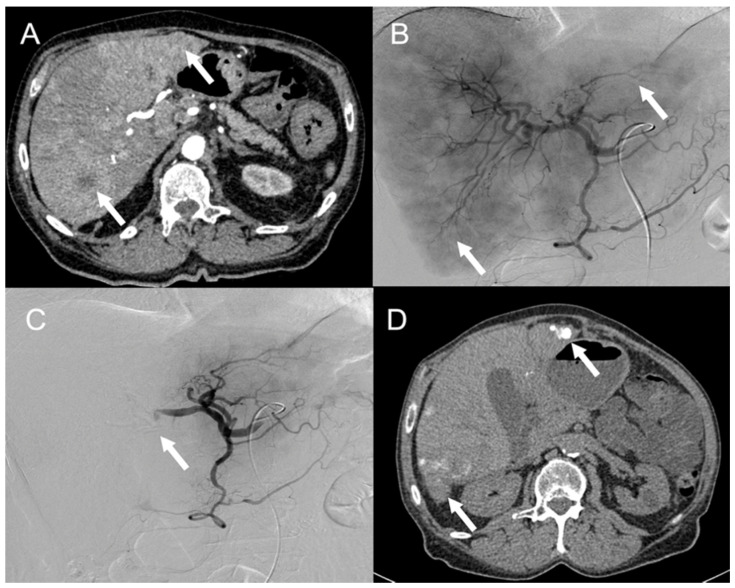
A 66-year-old patient with exclusively hepatic metastasis of a neuroendocrine tumor of unknown origin presented with important abdominal pain and carcinoid syndrome despite cold somatostatin analogues treatment. Preembolization contrast-enhanced arterial CT (**A**) and digital subtraction angiography (DSA) (**B**) show bilateral hypervascular liver metastases (arrows). DSA performed after a first TACE session in the right liver (**C**) shows complete devascularization of right liver lesions and contrast stagnation in the right branch of the hepatic artery (arrow). Postembolization CT performed 2 months after a second TACE session (**D**) performed on the left liver shows persistent Lipiodol retention in the treated metastasis (arrows).

While normal liver parenchyma is mainly vascularized by the portal vein, metastases are almost exclusively vascularized by the arterial hepatic system. This is particularly evident for NEN liver metastases showing typical arterial enhancement on cross-sectional imaging. Therefore, transarterial embolization (TAE) and transarterial chemoembolization (TACE) are particularly adapted to induce ischemic damage to target NEN metastases with a relative sparing of the surrounding normal liver parenchyma. TAE and TACE can be proposed as palliative treatments in patients with low-grade (G1 or G2) NENs with unresectable locally evolving or persistently symptomatic hepatic metastases despite systemic treatment mainly based on cold somatostatin analogues [48,49] (Table 1; Figure 4).

Previous resection of the primary tumor is not mandatory to propose TAE or TACE treatments [49]. TAE corresponds to catheterization of the hepatic artery and embolization using calibrated microparticles or ethiodized oil associated with gelfoam. TACE associates this procedure with the use of chemotherapeutic agents (usually doxorubicin, cisplatin, gemcitabine, or mitomycin C) based on the principle that ischemia of tumor cells increases sensitivity to chemotherapy [48,50]. TACE is performed either with an oily emulsion of chemotherapy (cTACE) or with drug-loaded beads (DEB-TACE). To improve treatment tolerance, one hepatic lobe is treated per session. The procedure can be repeated every 4 to 6 weeks as long as the patient meets the eligibility criteria for treatment and the treatment is effective in slowing tumor growth and reducing symptoms. In addition to general contraindications for vascular procedures (i.e., impaired hemostasis, allergy to iodinated contrast media), specific contraindications include portal vein thrombosis and bilio-enteric anastomosis. Patients with a tumoral burden greater than 75% of the liver parenchyma or who exhibit liver function impairment (bilirubin level ≥ 3 mg/dL, ascites) should be treated carefully, as they are at high risk of acute liver failure shortly after transarterial treatment [49,50]. Overall, 80–86% of patients will exhibit post-procedural symptoms, mostly in the form of a post-embolization syndrome (fever, nausea, vomiting, abdominal pain, and elevated liver enzymes) that should be anticipated and treated with analgesics, steroids, and hydration.

Major complications may occur in 3–17% of patients and include liver failure, ischemic cholecystitis, acute carcinoid syndrome, and liver abscess [49,50]. Symptomatic improvement is expected in 50–90% of patients [50]. Furthermore, 80–100% of patients will also show a radiologic response [50,51]. In responders, hepatic progression-free survival is over 10 months [52]. Currently, due to the lack of prospective studies, there is no clinical evidence supporting TACE over TAE in the treatment of NEN liver metastases [51]. Similarly, the place of TAE and TACE in comparison with radioembolization still needs to be clarified [48].

### 6.2. ^90^Y-Selective Internal Radiation Therapy (SIRT)

**Figure 5:**
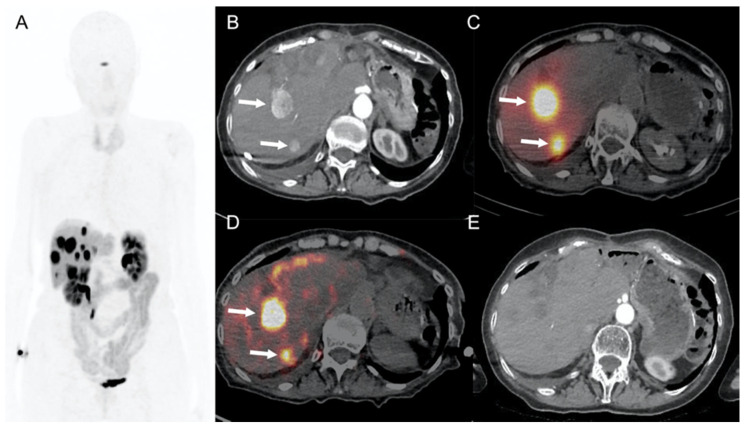
A 77-year-old woman with a previous history of surgically treated pancreatic grade 1 NEN (Ki67 = 1%) presented during follow-up with exclusive hepatic relapse. Multidisciplinary committee stated for ^90^Y-selective internal radiation therapy (SIRT) treatment. (**A**) Restaging anterior MIP of ^68^Ga-DOTATOC PET performed during follow-up; (**B**) arterial-phase contrast-enhanced CT obtained before SIRT; (**C**) pretreatment scintigraphy (axial SPECT/CT) performed during work-up after injection of macroaggregated albumin labeled with ^99m^Technetium showing intense uptake by metastasis (arrows) and slight uptake by normal liver parenchyma; (**D**) posttreatment PET/CT (axial slice) confirming the intense uptake by liver metastasis (arrows) perfectly concordant with pretreatment scintigraphy; and (**E**) 6-month CT (arterial phase, axial slice) after SIRT showing tumoral involvement and treatment efficacy.

The use of selective internal radiation therapy (SIRT), also known as radioembolization, has greatly improved in the last two decades, particularly in patients with hepatocellular carcinoma (HCC), as a therapeutic alternative to TACE [53,54,55]. SIRT has also been proposed to NEN patients presenting with advanced disease as a therapeutic choice for unresectable liver metastases [56,57] (Table 1; Figure 5).

SIRT allows the release of a radioactive source carried by small embolization agents (i.e., spheres) injected transarterially through a catheter into the hepatic arterial system [50]. 90-Yttrium (^90^Y) is the most widely used therapeutic radioisotope for hepatic radioembolization. ^90^Y is a pure beta-emitter (electron) with an average energy of 0.94 MeV [58] and a half-life of 64 h (2.67 days). The maximum and average ranges of beta emission in tissues are 11 and 2.5 mm, respectively [59]. The use of 166-Holmium (^166^Ho) has also been proposed, but it is still under evaluation. Concerning the available embolization agents, there are two different types according to the nature and the activity per sphere: resin spheres (SirSphere(R), 20 to 60 µm diameter; 50 Bq per particle) and glass spheres (TheraSphere(R), 20 to 30 µm diameter; 2500 Bq per particle). The therapeutic action of hepatic radioembolization is twofold because it acts as a radiation therapy agent and, to a lesser extent, as a transarterial embolization treatment. NEN liver metastases are hypervascularized lesions with anarchic vasculature and highly permeable vessels. To be efficient, radioactive spheres need to be conducted from the arterial blood flow to the tumor terminal capillaries being sized less than the sphere itself. As a result, most of the radioactivity will be deposited on the target tumor without affecting systemic circulation. In clinical practice, selective (left/right liver) or hyperselective (liver segment) arterial catheterization is possible in relation to tumor topography and therapeutic strategy.

Before SIRT, a multidisciplinary work-up is performed to predict and optimize radiation delivery to both the target tumor and the other at-risk organs, including the lungs. The therapeutic procedure is simulated by injecting 150 MBq of albumin macroaggregates (MAAs) radiolabeled with 99m-technetium (^99m^Tc, gamma emitter, 140 keV, 6 h half-life) under the same conditions as SIRT. The work-up is performed up to 4 weeks before SIRT and provides a good idea of MAA liver distribution, thus providing real patient-tailored dosimetry along with precise calculation of the activity of ^90^Y that will be injected. The minimum absorbed dose cutoff proposed for NENs to yield tumoral response is 100–150 Gy [60]. Moreover, the work-up allows the detection and estimation of any hepatopulmonary shunt (HPS) by gamma-camera imaging to limit any unintentional irradiation to the lung; in fact, any major HPS significantly increases the risk of radiation-induced pneumonitis and may contraindicate SIRT [60]. Post-SIRT imaging is performed by gamma-camera (bremsstrahlung emission) or PET (annihilation) within the day following SIRT to assess tumoral targeting and the absence of extrahepatic deposits of the radioactive spheres. To avoid radiation pneumonitis, the predicted lung dose radiation should not exceed 30–50 Gy. Radioembolization-induced liver disease (REILD) may also occur after SIRT, especially in patients with pre-existing liver disease, those who have received previous chemotherapies, and those with extensive liver tumor burden. Elevated bilirubinemia, intrahepatic biliary duct dilatation, portal vein obstruction, and high HPS are contraindications to SIRT.

To assess the value of ^90^Y microspheres in the management of unresectable liver metastases secondary to NENs, Jia et al. [56] recently performed a meta-analysis including 870 patients from 11 studies and 7 abstracts. Approximately 20% of patients underwent TAE or TACE prior to SIRT. Among all patients, the median disease control rate at 3 months after SIRT was 86%, and the median survival was 28 months. According to the primary tumor origin, the patient survival rate after SIRT was 56, 31, and 28 months for small intestine carcinoid tumors, pancreatic NENs, and primary NENs of unknown origin, respectively. Depending on the grade of the tumor, the survival rate was 71 months for grade 1 NENs, 56 months for grade 2 NENs, and 28 months for grade 3 tumors. The median disease control rate was 86% 3 months after SIRT. The most common secondary effects were abdominal pain (median, 32.6%), nausea/vomiting (median, 32.5%), and asthenia (median, 30.4%). Moreover, four cases of radiation gastritis, two cases of duodenal ulcer, on case of radiation cholecystitis, and one case of disease secondary to liver failure were also reported. Similar results were reported by Frilling et al. [61] in a meta-analysis including 23 studies showing a mean disease control rate of 88% and a median progression-free survival of 41 months after SIRT. Overall, it appears that ^90^Y microsphere SIRT could be considered as a valid and safe therapeutic alternative for unresectable liver metastases of NENs, with an improved survival rate and tumor response. Moreover, SIRT can be performed after unsuccessful TAE or TACE and to decrease symptoms related to carcinoid syndrome in patients with secreted NENs. Radioembolization can also be safely performed after systemic radionuclide treatments such as peptide receptor radionuclide therapy (PRRT), with rare occurrence of radioembolization-induced liver disease. Braat et al. [62] reported the results of a retrospective analysis of forty-four patients with NENs who underwent a total of 58 radioembolization procedures (55% whole liver treatments) at a median of 353 days after prior PRRT. An objective response rate of 16% and a disease control rate of 91% were observed after 3 months. Moreover, 65% (15/23) of symptomatic patients showed a clinical response after 3 months. For the entire study population, a median overall survival of 3.5 years after radioembolization was found. Within 3 months, clinical and grade 3–4 hematological toxicities occurred in 26% and less than 10% of treated cases (apart from lymphocytopenia occurring in 42%), respectively. Finally, radioembolization-related complications occurred in 5% of patients, and fatal radioembolization-induced liver disease occurred in one patient.

The prognostic factors affecting patient response and survival after ^90^Y-radioembolization in patients with unresectable NEN liver metastases were investigated by Saxena et al. [63] by analyzing 48 patients at their institution. Five prognostic factors were associated with improved survival: complete/partial response, low hepatic tumor burden, female sex, well-differentiated tumor, and absence of extrahepatic disease. Moreover, female sex, tumor differentiation, and low hepatic tumor burden were associated with a complete/partial response after SIRT.

### 6.3. Targeted Radionuclide Therapy

Targeted radionuclide therapy represents the most common example of patient-specific therapies based on the “image and treat” approach” [64]. It allows the use of molecular vectors labeled either with diagnostic or with therapeutic radionuclides, and it is one of the most interesting associations in patients with NENs is ^68^Ga-DOTATATE and ^177^Lu-DOTATATE, with very promising results. The phase 3 NETTER-1 trial is to date, the only prospective multicenter, randomized controlled trial comparing ^177^Lu-DOTATATE (four cycles of intravenous injection of 7.4 GBq ^177^Lu-DOTATATE per cycle every 8 weeks) versus high-dose SSA in advanced progressive low-grade midgut NENs (n = 229) [65]. The progression-free survival at 20 months was 65.2% (95%, CI: 50.0–76.8%) in the PRRT arm and 10.8% (95% CI: 3.5–23.0%) in the control arm. It is noteworthy that the effect in tumor control was accompanied by a clinically meaningful effect on the quality of life of patients with somatostatin-receptor-positive midgut NENs [66]. The NETTER-2 trial is ongoing to determine whether ^177^Lu-DOTATATE PRRT in combination with long-acting octreotide prolongs progression-free survival in GEP-NET patients with high proliferation rate tumors (G2 and G3) when given as a first-line treatment compared with treatment with high dose (60 mg) long-acting octreotide (NCT03972488).

Although PRRT remains well-tolerated with limited sub-acute toxicity that is commonly mild and self-limiting, long-term side effects are mainly radiation nephropathy and persistent hematological dysfunction [65]. Intra-arterial administration of PRRT has also been explored. The therapeutic effectiveness of DOTATOC labeled with the therapeutic beta emitters ^90^Y and ^177^Lu directly injected into the hepatic artery has been evaluated in patients with NEN liver metastases [67]. The high rate of objective response in patients treated with arterial infusion of ^90^Y/^177^Lu-DOTATOC compares favorably with intravenous PRRT, warranting further investigations of this alternative approach in terms of therapeutic effectiveness and long-term toxicity. However, only selected patients with tumors of limited anatomic distribution could be eligible and truly benefit from intra-arterial PRRT. Moreover, such a therapeutic option is still not available on a large scale.

## 7. Conclusions

In recent years, considerable progress has been made in the therapeutic strategy for NENs, underlining that a multidisciplinary approach remains essential in the therapeutic discussion of patients with metastatic NENs. Considering their long survival, patients are likely to receive multiple treatments, although the optimal therapeutic strategy has not yet been completely defined. In patients with NENs, liver metastases for whom hepatic surgery is contraindicated, percutaneous or intra-arterial treatments are safe and effective options for achieving disease control. In selected patients, they allow the improvement of clinical symptoms and biological abnormalities related to tumor secretion. However, toxicity and quality of life are also important elements in therapeutic decisions and must be considered for each single patient. Prospective studies are necessary to define the best treatment combination, including systemic and local options.

## Figures and Tables

**Table 1:** Clinical indications and contraindications of the main liver-directed therapy procedures for neuroendocrine metastasis treatment, including interventional radiology and nuclear medicine options.

Treatment	Main Indications	Contraindications	Advantages	Disadvantages
Percutaneous ablation	Oligo-metastatic disease (less than 3–5 metastases) Oligo-progressive disease (1–2 metastases not responding to systemic treatments)	Irreversible coagulative disorders Contraindications to sedation or general anesthesia Bilio-enteric anastomosis/history of sphincterotomy Dilatation of intra-hepatic biliary tree due to biliary strictures Cardiac arrythmia in case of electroporation	Minimally invasive Relative fast post-operative recovering phase Can be repeated	Useful for a limited burden of disease only
Transarterial embolization (TAE) Transarterial chemo-embolization (TACE)	Unresectable hepatic metastatic disease or not suitable for thermal ablation Disease progression or persistent symptoms despite cold somatostatin analogues therapy	Portal vein thrombosis Bilio-enteric anastomosis/history of sphincterotomy Liver involvement >75% Impaired hepatic function (bilirubin level ≥3 mg/dl, ascites) Allergy to contrast media Irreversible coagulative disorders	Treat a large and diffuse disease Can be repeated TACE provides a combined ischemic and chemotherapy effect on large and/or diffuse disease	Frequent post-embolization syndrome TAE provides an ischemic effect only Needs 6–12 h of in-bed stay after treatment due to the arterial femoral access
Selective Internal Radiation Therapy (SIRT) or radioembolization		Pre-existing liver disease, including patients who have previously received chemotherapies Impaired hepatic function (bilirubin level ≥3 mg/dl, ascites) Greater than 20% lung shunting of the hepatic artery blood flow determined during the work-up Pre-assessment angiogram that demonstrates abnormal vascular anatomy that would result in significant reflux of hepatic arterial blood to the stomach, pancreas, or bowel	Better tolerance profile compared with TAE and TACE	Needs two separate vascular procedures (work-up and treatment) Needs 6–12 h of in-bed stay after treatment due to the arterial each femoral access Needs well-organized institutional protocols
177Lu-Peptide receptor radionucl. therapy (intra-arterial PRRT)	Clinical trials	Clinical trials Negative somatostatin receptor imaging	Clinical trials	Clinical trials Not an option in most centers
